# Evaluation of 2-methyl-4-isothiazolin-3-one-induced human pulmonary toxicity using integrated air-liquid-interface and a lung-on-chip

**DOI:** 10.1186/s12931-026-03733-z

**Published:** 2026-05-27

**Authors:** Sohyun Park, Lea L. de Maddalena, Saskia Schmid, Arunima Sengupta, Chang Gyu Woo, Nina Hobi, Young-Jae Cho

**Affiliations:** 1 Division of Pulmonary and Critical Care Medicine, Department of Internal Medicine, Seoul National University College of Medicine, Seoul National University Bundang Hospital, Seongnam-si, Gyeonggi-do Republic of Korea; 2 Department of Health Science and Technology, Graduate School of Convergence Science of Technology, Seoul National University, Suwon-si, Gyeonggi-do Republic of Korea; 3 AlveoliX AG, Swiss Organs-on-Chip Innovation, Bern, Switzerland; 4 Alexis Technologies AG, Bern, Switzerland; 5 School of Mechanical Engineering, Korea University of Technology & Education (KOREATECH), Cheonan-si, Chungcheongnam-do Republic of Korea

**Keywords:** Pulmonary toxicity, Lung-on-chip, 2-methyl-4-isothiazolin-3-one, Humidifier disinfectant, Air-liquid-interface

## Abstract

A 3:1 disinfectant composed of 5-chloro-2-methyl-4-isothiazolin-3-one (CMIT) and 2-methyl-4-isothiazolin-3-one (MIT) has been widely used in water-based household products such as detergents, toothpaste, and cosmetic applications. In the United States, it was classified as a pesticide in 1998 and designated as a Category 2 dermal-toxicity substance for cosmetics. Several studies in the Republic of Korea have reported that components of humidifier disinfectants (HDs) such as polyhexamethylene guanidine (PHMG), CMIT, and MIT cause chronic pulmonary conditions. However, its toxicity is still controversial because of the discrepant results between human cases and animal experiments. We recapitulate the in vivo characteristics of the pulmonary epithelial cells and introduce an air-liquid interface (ALI) system to replicate acute inhalation injury. To assess compound-specific toxicity in the human airway, NHBE cells were first differentiated under ALI conditions and then exposed to MIT under submerged conditions. In parallel, ^AX^Barrier-on-Chip (AlveoliX AG) technology integrated with the Cloud α AX12 (Vitrocell Systems GmbH) allowed us to mimic human lung physiology more realistically. In this study, this inhalation platform was utilized to nebulize MIT onto primary cell-derived immortalized alveolar epithelial cells (^AX^iAECs) co-cultured with human lung microvascular endothelial cells (hLMVECs) seeded onto the AX12. Additionally, PHMG was used as a positive control to compare MIT toxicity levels. Using this approach, significant barrier disruption and cytotoxicity were observed when MIT was introduced into the alveolar lung-on-chip. Overall, we confirmed MIT-induced toxicity in both bronchial and alveolar epithelial models, highlighting the importance of multiscale in vitro systems for inhalation toxicology.

**Supplementary Information:**

The online version contains supplementary material available at 10.1186/s12931-026-03733-z.

## Background

In South Korea, humidifiers are widely used to maintain indoor humidity and prevent respiratory diseases. However, without regular cleaning, microorganisms can proliferate in the water. To address this, chemical companies previously recommended adding disinfectants that were considered safe at the time to inhibit germs, molds, and algae. Despite strict recommendations to prevent adverse effects, the use of humidifier disinfectant (HDs) has led to severe health consequences. In South Korea, humidifier disinfectant-associated lung injury (HDLI) refers to lung conditions such as interstitial pneumonitis and widespread lung fibrosis, which have been linked to the use of chemical HDs since 1994 and prompted government investigations and compensation programs for affected individuals [1]. According to the Humidifier Disinfectant Damage Support Portal developed by the Korea Environmental Industry & Technology Institute (KEITI), 1,886 deaths in South Korea were attributed to the inhalation of HDs over the 10-year period preceding its report in 2024 [2]. The substances found to be responsible for these health issues most commonly include polyhexamethylene guanidine (PHMG), oligo(2-(2-ethoxy)ethoxyethyl) guanidinium chloride (PGH), and a mixture of 5-chloro-2-methyl-4-isothiazolin-3-one (CMIT) and 2-methyl-4-isothiazolin-3-one (MIT). In response to the toxicity of these chemicals, the Korea Center for Disease Control and Prevention (KCDC) declared HDs to be a potent cause of lung injury, leading to a nationwide ban on their usage in 2011 [3, 4]. From the time of commercial introduction of the first HD in 1994 until the government-mandated recall in November 2011 due to their toxic nature, more than 9.5 million units of over 25 different consumer products were sold. According to a 2014 report by the KCDC Lung Injury Committee of the Ministry of Health and Welfare, the causality of substances such as PHMG and PGH in the incidence of lung injuries was confirmed through epidemiological investigations and animal inhalation toxicity tests [5, 6]. MIT and CMIT are synthetic biocides widely utilized across various industries for their broad-spectrum antimicrobial properties. Despite their low toxicity and favorable environmental profile, these compounds have been implicated in contact hypersensitivity and allergic contact dermatitis, raising concerns about their safety in consumer products [7, 8]. Regulatory actions in 2016 banned their use in leave-on products and restricted MIT concentrations in rinse-off products in Europe [9].

Park et al. (2017) conducted an epidemiological case series analysis of 530 patients with HDLI, revealing a significant association with multiple chemical disinfectants (PHMG, PGH, and CMIT/MIT). Among the 36 patients who had exclusively used CMIT/MIT-based products, three (8.3%) were diagnosed with HDLI by a multidisciplinary panel using clinical, radiologic, and, when available, pathologic criteria. Although this proportion is relatively low, it was evidence that CMIT/MIT alone can induce clinically significant pulmonary damage [1].

Song et al. (2022) investigated the toxicity of CMIT/MIT in the lungs by examining its effects through intranasal and intratracheal instillation in mice. Their biodistribution study revealed that CMIT/MIT rapidly spreads through the respiratory tract, with high levels detected in the lungs shortly after administration. Mice exposed to CMIT/MIT exhibited lung injury, which was confirmed by increased inflammatory cells in bronchoalveolar lavage fluid (BALF) and lung tissue damage. This study highlighted that lung injuries were dose-dependent, with intratracheal exposure causing more severe toxicity than intranasal exposure [10].

MIT, widely used for its antimicrobial properties, has been shown to induce apoptotic cell death, oxidative stress, and membrane damage in the BEAS-2B human bronchial epithelial cell line via matrix metalloproteinase activation and demonstrated significant cytotoxicity even at relatively low concentrations. Furthermore, MIT triggers cell death and inflammatory responses, partly through DNA damage, in the liver epithelium, illustrating its broader toxicity across multiple human cell types [11, 12].

While there has been extensive research on the health effects of PHMG in vivo, relatively little research on the effects of PHMG at a cellular level has been reported. Recent studies using the Cloud α AX12 platform have shown that PHMG aerosol exposure on human lung cells causes severe toxicity and barrier disruption along with heightened inflammation [13].

Despite valuable insights from human case reports and animal experiments, critical gaps remain—particularly regarding the precise cellular mechanisms of disinfectant-induced toxicity under realistic exposure conditions. Furthermore, animal models, while indispensable, may not fully replicate human lung architecture or immunology.

In this study, 96-well plates were defined as a two-dimensional (2D) culture system, whereas Transwell and alveolar lung-on-chip platforms were defined as three-dimensional (3D) systems due to their more tissue-like spatial organization and multicellular interactions. Consequently, we used Transwell to represent the bronchial epithelial environment and the ^AX^Barrier-on-Chip to model the alveolar region. The latter was integrated with the Cloud α AX12 platform, a next-generation inhalation system that combines aerosolization with ALI conditions to simulate a physiologically relevant alveolar microenvironment. In this system, epithelial–endothelial co-culture was used to better recapitulate the air–blood barrier than an epithelial-only model, as epithelial–endothelial crosstalk is known to influence barrier function and inflammatory responses to external stimuli [14]. This approach enables a more realistic evaluation of MIT—a HD component—by mimicking human exposure scenarios. By bridging advanced in vitro technology and real-world applications, our study highlights the importance of assessing aerosolized chemical toxicants in physiologically relevant environments, which will contribute to improved risk assessments and enhanced public health strategies for chemical exposure mitigation.

## Materials and methods

### Cell culture maintenance

Primary normal human bronchial epithelial (NHBE) cells were sourced from Lonza (Lonza, Walkersville, MD, USA). The cells were cultivated in T75 cell culture flasks with BEGM™ Bronchial Epithelial Cell Growth Medium BulletKit™ (Lonza) at 37 °C and 5% CO_2_. Passage 3 NHBE cells were seeded atop Transwells (Costar, Corning, NY, USA) coated with 0.3 mg/mL Collagen type IV from human placenta (Sigma-Aldrich, St. Louis MO, USA), which was diluted 1:1 in Dulbecco’s phosphate-buffered saline (DPBS), at a cell density of 5 × 10^4^ cells/well. The seeded Transwells were then submerged in PneumaCult-Ex Plus (PnC-Ex-PLUS) media (StemCell, Tukwila WA, USA) for four days to allow the epithelium to attach and reach 90% confluence. Upon reaching confluency, the apical media was removed and the basal media was replaced with PneumaCult-ALI medium (StemCell). Human Umbilical Vein Endothelial Cells (HUVEC) (Lonza) were cultivated in T75 cell culture flasks with EGM™-2 Endothelial Cell Growth Medium-2 BulletKit™ (Lonza). Low-passage HUVECs were seeded in Transwells at a density of 1 × 10^5^/well. For the co-culture model, the bottom was coated with Fibronectin™ (Sigma-Aldrich) diluted into 0.01%. NHBE cells were seeded on the top and cultured at an ALI for four weeks as previously described. The Transwells were inverted on a petri dish, and the basolateral side was seeded with HUVEC cells. They were kept inverted for two hours to allow cell adhesion and then returned to their original orientation for continued culture. The co-culture model was stabilized for four days with a 1:1 mixture of EGM and ALI media.

The ^AX^iAECs used in this study were obtained from AlveoliX (Switzerland). These cells were cultured according to the manufacturer’s instructions. The cell model has been previously described and characterized. Expression of markers characteristic of physiologically relevant AT I and AT II cells has been confirmed [13, 15, 16]. On the AX12, the AX co-culture Biomodel was cultured according to the manufacturer’s instructions, using the optimized co-culture medium, ^AX^E2 Alveolar Barrier Medium (^AX^E2-ABM) supplemented with 1% penicillin streptomycin, by performing a medium exchange every two to three days. After a barrier was formed, the cells were switched to ALI and cyclic stretch.

### Liquid MIT treatment of Transwell

After four weeks of ALI differentiation, NHBE/HUVEC co-cultures in Transwell inserts were exposed under submerged conditions. MIT stock solutions were prepared in a 0.9% NaCl solution and subsequently diluted in an ALI medium to obtain final concentrations of 1–250 µg/mL. For each Transwell, 200 µL of ALI medium containing MIT was added to the apical compartment and 500 µL of ALI medium was added to the basolateral compartment.

### ^AX^Barrier-on-Chip System

Similar to the previously described ^AX^Lung-on-Chip system, we also employed the ^AX^Barrier-on-Chip system (AlveoliX AG, Switzerland) [13, 16]. The system consists of a consumable, the AX12, two electro-pneumatic devices, and the ^AX^Exchanger and ^AX^Actuator, which are connected to the ^AX^Barrier-on-Chip consumable through an interface platform (^AX^Dock). AX12 with seeded cells is hereafter referred to as alveolar lung-on-chip. Cell seeding and medium exchange were performed according to the established in-house protocol for the AlveoliX. For medium exchange and sample collection, AX12 is positioned within the ^AX^Dock in aseptic conditions and the corresponding command on the ^AX^Exchanger was used. To initiate the 3D breathing motion of the lung, the AX12 plate is positioned within the ^AX^Dock in the incubator. Three-dimensional cyclic stretching (schematic shown in Fig. 2A) was then initiated through a touchscreen control on the ^AX^Actuator, identical to the ^AX^Breather, which deflects the micro-diaphragm by generating negative cyclic pressure.

### Cloud α AX12 exposure

The Cloud α AX12 platform (developed jointly by Vitrocell GmbH and AlveoliX AG) was used for exposure of cell cultures to control nebulized aerosols at ALI. For the 3D alveolar model, the AX co-culture Biomodel was seeded on the AX12 and cultured under breathing conditions. MIT was diluted in 0.9% NaCl solution with a final volume of 300 µL. The concentrations of nebulized MIT were 1, 10, 25, 50, 100, and 250 µg/mL (Sigma-Aldrich, Ref # 725765), with corresponding expected depositions of 0.002 µg/cm², 0.021 µg/cm², 0.053 µg/cm², 0.106 µg/cm², 0.212 µg/cm², and 0.53 µg/cm², respectively. As a positive control, PHMG (Boc Sciences, CAS #89697-78-9) was diluted in 0.9% NaCl solution to a final volume of 300 µL, resulting in an initial concentration of 1285 µg/mL. The final deposition measured on the cells was 2.7 µg/cm². Exposure was performed at day 21. Cytotoxicity and viability were measured at 4-, 24-, and 48-hours post-treatment for the alveolar lung-on-chip. PHMG (1280 µg/mL, expected deposition: 2.72 µg/cm²) served as the positive control while 0.9% NaCl was used as the vehicle. Technical replicates included four wells for each condition, with endpoints at 4, 24, and 48 h for trans-epithelial electrical resistance (TEER) and cytotoxicity assessments, as well as RNA isolation and immunofluorescence imaging at the final timepoint.

Before proceeding to the alveolar lung-on-chip, we selected concentrations by testing in a 2D alveolar model via a 96-well plate. The ^AX^iAECs were seeded and then exposed to various MIT concentrations via the platform. The concentrations of nebulized MIT were 1, 10, 25, 50, 100, and 250 µg/mL (Sigma-Aldrich, Ref # 725765). Cytotoxicity and viability were measured 24 h post-treatment at these concentrations to determine suitable levels for the alveolar lung-on-chip. Based on the results from the 2D alveolar model, low, medium, and high MIT concentrations were established for use in the alveolar lung-on-chip.

### Bright-field imaging

Bright-field images of Transwell cultures were acquired using an Olympus CKX53 inverted microscope (Olympus Corporation, Japan) equipped with a 4× objective and controlled by iSolution LITEx64 software. Images were captured with an exposure time of 10 ms, gain 1, at a resolution of 1536 × 1024 pixels and 24-bit color depth (96 dpi).

### Cell viability

In the Transwell model, the cells were then incubated in a medium containing MIT for 24 h. Cell Counting Kit-8 (CCK8, Dojindo, Kumamoto, Kyushu, Japan) was added to each well and further incubated for 1 h at 37 °C. The absorbance was quantified using a SpectraMax iD3 Multi-Mode Microplate Reader (Molecular Devices, San Jose, CA) at 450 nm.

On chip, the PrestoBlue Cell Viability Assay (A13261, Life Technologies) was performed on cells cultured on the AX12 according to the manufacturer’s instructions. Briefly, 1X PrestoBlue solution was prepared by diluting the 10X reagent in the cell culture medium. After removing the old medium, 70 µL of the 1X PrestoBlue solution was added to each well of the AX12 chip, and the cells were incubated at 37 °C for two hours. Following incubation, the supernatant was gently homogenized and 80% of the total volume was transferred to a black 96-well plate. Absorbance was measured using a Tecan plate reader at 555 nm. A blank control (1X PrestoBlue solution without cells) was included, and background values were subtracted from each sample for analysis. The absorbance values were then normalized to the control group.

### Immunofluorescence staining and imaging on transwell

The cells were fixed in 4% paraformaldehyde in DPBS at room temperature (RT) and were permeabilized with 0.1% Triton X-100 in DPBS for 15 min at RT. Subsequently, the cells were blocked with 3% bovine serum albumin (BSA) in DPBS for 30 min at RT and reacted overnight with primary antibodies in 1% BSA; E-Cadherin and MUC5AC. The first antibodies used for the study are rabbit anti-E Cadherin antibody (1:200 dilution; C# ab40772, Abcam, Cambridge) and mouse anti-MUC5AC antibody (1:250 dilution; C#MA5-12178, Invitrogen, Thermo Fisher Scientific, Waltham, MA). Additionally, to further characterize epithelial phenotypes, the cells were stained with a ciliary marker (Alexa Fluor 647-conjugated anti-beta IV Tubulin; 1:200 dilution; C# ab204023, Abcam) and a club cell marker (rabbit anti-CC10; 1:100 dilution; C# ab317665, Abcam). The primary antibodies were diluted in 1% BSA in DPBS and incubated overnight at 4 °C. Secondary antibodies were used as follows: Donkey anti-mouse Alexa Fluor 488 (1:250 dilution; C# ab150105, Abcam) and donkey anti-rabbit Alexa Fluor 647; C# ab150075, Abcam) were diluted in 1% BSA in DPBS and incubated for 2 h at RT. Nuclei were stained with Hoechst (1:1000 dilution; C# 33342, Invitrogen). Cell morphology and actin‑rich structures were visualized using CellTracker™ Red CMTPX Dye (1 drop/mL; C# R37112, Invitrogen). Imaging was conducted using a Leica Thunder Imager (Leica Microsystems GmbH, Germany) and a 40× water-immersion objective. Images were acquired at a resolution of 3072 × 2048 pixels with 16-bit depth, with exposure times maintained within 2–12 ms and gain set to 1 for all channels. The integrated LED illumination intensity was optimized between 8% and 14% to minimize photobleaching. Images were processed post-acquisition using Leica Application Suite X (LAS X, Leica Microsystems) software. Only linear adjustments of brightness and contrast were applied uniformly across all images; no deconvolution or additional computational clearing was performed.

### Immunofluorescence staining and imaging for alveolar lung-on-chip

Cells cultured on the AX12 were initially fixed at the endpoint (48 h) using Image-It Fixative Solution™ (R37814, Invitrogen) as per the manufacturer’s instructions and thoroughly washed with 1X DPBS without calcium and magnesium utilizing the “Medium exchange” function on the ^AX^Exchanger to fix cells within the basal chamber. After fixation, chips were disassembled using the ^AX^Disassembly tool (AlveoliX, Switzerland). Cells were permeabilized with 0.1% Triton X-100 in DPBS for 15 min at RT, followed by incubation in blocking buffer (2% BSA in DPBS) for one hour at RT and then stained with the following antibodies: Hoechst (1:2000, Invitrogen #33342), Anti-Zonula Occludens 1-488 conjugated (1:100, Thermo Fisher #339188), and Anti-CD31-567 conjugated (1:100, Cell Signaling #61255), followed by overnight incubation. Stained membranes were mounted between two glass coverslips using a mounting medium (Sigma-Aldrich, #F6182). Confocal representative images were acquired using a Zeiss LSM 980 microscope (Carl Zeiss Microscopy GmbH, Germany). Images were acquired with an oil-based immersion objective at a resolution of 1024 × 1024 pixels, gain set to 1 for all channels. Images were processed using the Zeiss Zen Black software version 3.11.

### ELISA

In the Transwell model, to measure protein levels by Enzyme-Linked Immunosorbent Assay (ELISA), after 24 h exposure the apical medium was gently aspirated, and 100 µL of pre-warmed ALI medium was added to the apical compartment for 10 min at 37 °C to collect apical secretions. The resulting apical wash and basolateral medium were collected, and cytokine levels were quantified using a Human Quantikine ELISA Kit (R&D Systems, Minneapolis, MN). The intensity of the produced color was measured at 450 nm using a SpectraMax iD3 Multi-Mode Microplate Reader (Molecular Devices, San Jose, CA).

### Multiplex

Cell culture supernatants from the AX12 support were collected by adding 100 µL of pre-warmed PBS per well on the apical side and gently mixing. The supernatant was then centrifuged and stored at -80 °C until analyzed. Secreted proteins were quantified with a highly sensitive ProcartaPlex multiplex panel (Invitrogen, MAN0024966) based on Luminex xMAP technology. The analysis was performed with Luminex xPONENT software (Luminex, Texas, USA). Standard curves of known concentrations of all cytokines were used to determine the concentration of the secreted proteins in pg/ml. Samples with a concentration below the lowest standard but above the background were assigned the concentration of the lowest standard and included in the analysis.

### TEER measurement

To assess barrier formation, TEER measurements were conducted every two days on the AX12, starting 48 h post cell seeding, as previously described [16]. In brief, a 96-well plate electrode (STX100MC96; World Precision Instruments) along with an Epithelial Volt/Ohm Meter (EVOM3; World Precision Instruments) were utilized for this purpose. Prior to each measurement, electrodes were sterilized with 70% ethanol for five minutes, followed by rinsing with distilled water for five minutes at RT. Readings were taken using the “TEER measurement” feature on the ^AX^Exchanger. For cells maintained in ALI, pre-warmed sterile 1X DPBS was added 15 min before measurement and re-equilibrated after TEER readings. Background TEER values (Ohms) were obtained from wells without cells. The final TEER readings (Ohm.cm²) were calculated by subtracting the background resistance and multiplying the result by the surface area of the cell culture well in AX12. To express the data as a relative change, these values were then normalized to the TEER before exposure (0 h).

### LDH cytotoxicity assay

On chip, apical supernatants collected from individual AX12 wells were examined for lactate dehydrogenase (LDH) release. A LDH-Glo cytotoxicity assay kit (Promega, #J2381) was utilized following the manufacturer’s instructions. All LDH measurements were performed in technical duplicates. The respective cell culture medium was used as a “blank” control and subtracted from each sample. Subsequently, the samples were normalized to the untreated control. Luminescence readings were obtained using a microplate reader (Tecan Reader M1000).

### RT-qPCR analysis

Gene expression was analyzed by reverse transcription–quantitative polymerase chain reaction (RT-qPCR). RNA was isolated using a Direct-zol™ RNA Microprep kit (Zymo Research, Switzerland) according to the manufacturer’s protocol. The RNA concentration was determined using a NanoDrop™ Spectrophotometer (Thermo Fischer Scientific, Switzerland). For cDNA synthesis, total RNA was reverse transcribed using a GoScript™ Reverse Transcriptase kit (Promega, Madison, WI, USA; Cat# A2791) according to the manufacturer’s instructions. The reverse transcription was carried out at 42 °C for one hour, followed by enzyme inactivation at 70 °C for 15 min. RT-qPCR was conducted for GAPDH, Interleukin (IL) -6, IL-1β, and IL-13. All experiments were carried out using a SensiFAST™ SYBR Lo-ROX Kit (Bioline, London) for target gene amplification according to the manufacturer’s instructions. All reactions were carried out in triplicate. The PCR amplification was carried out on a ViiA 7 Real-Time PCR System (Applied Biosystems, Thermo Fisher Scientific) under the following conditions: initial denaturation at 95 °C for two minutes, followed by 40 cycles of 95 °C for five seconds and 60 °C for 30 s. A melting curve analysis was performed to verify the specificity of the amplification. Data were analyzed using the comparative Ct method to quantify relative gene expression levels. Relative expression was quantified using the comparative cycle threshold (2^−ΔC^_t_) method, with GAPDH as the reference gene. The primer sequences are provided in Table 1.

**Table 1:** Primer sequences

Primer name	Type	Sequence (5’>3’)
IL-6 F	F	GGT ACA TCC TCG ACG GCA TCT
IL-6 R	R	GTG CCT CTT TGC TGC TTT CAC
IL-1β F	F	GCA CGA TGC ACC TGT ACG AT
IL-1β R	R	CAC CAA GCT TTT TTG CTG TGA GT
IL-13 F	F	GTG TCT GAA GCA GCC ATG GCA G
IL-13 R	R	CAA CAC GCA GGA CAG GTA CAG
GAPDH F	F	GTC TCC TCT GAC TTC AAC AGC G
GAPDH R	R	ACC ACC CTG TTG CTG TAG CCA A

### Statistical analysis

All data are presented as mean ± SEM. Statistical analyses were performed using GraphPad Prism 10. The data distribution and variance homogeneity were evaluated. If variances were homogeneous, an ordinary one-way ANOVA was performed followed by Dunnett’s multiple comparisons test versus the control. If variances were heterogeneous (Brown–Forsythe *p* < 0.05), Welch’s ANOVA with Dunnett’s T3 post hoc test (not assuming equal variances) was applied. For graphical annotations, statistical significance is indicated as *P* < 0.05 (*), *P* < 0.01 (**), *P* < 0.001 (***), and *P* < 0.0001 (****), and exact P values are provided in supplementary Fig. 2. In this study, n denotes the number of replicate wells (technical replicates) within a single experiment and N denotes the number of independent experiments (biological replicates).

## Results

### MIT induced cytotoxicity in a bronchial airway co-culture model

**Fig. 1:**
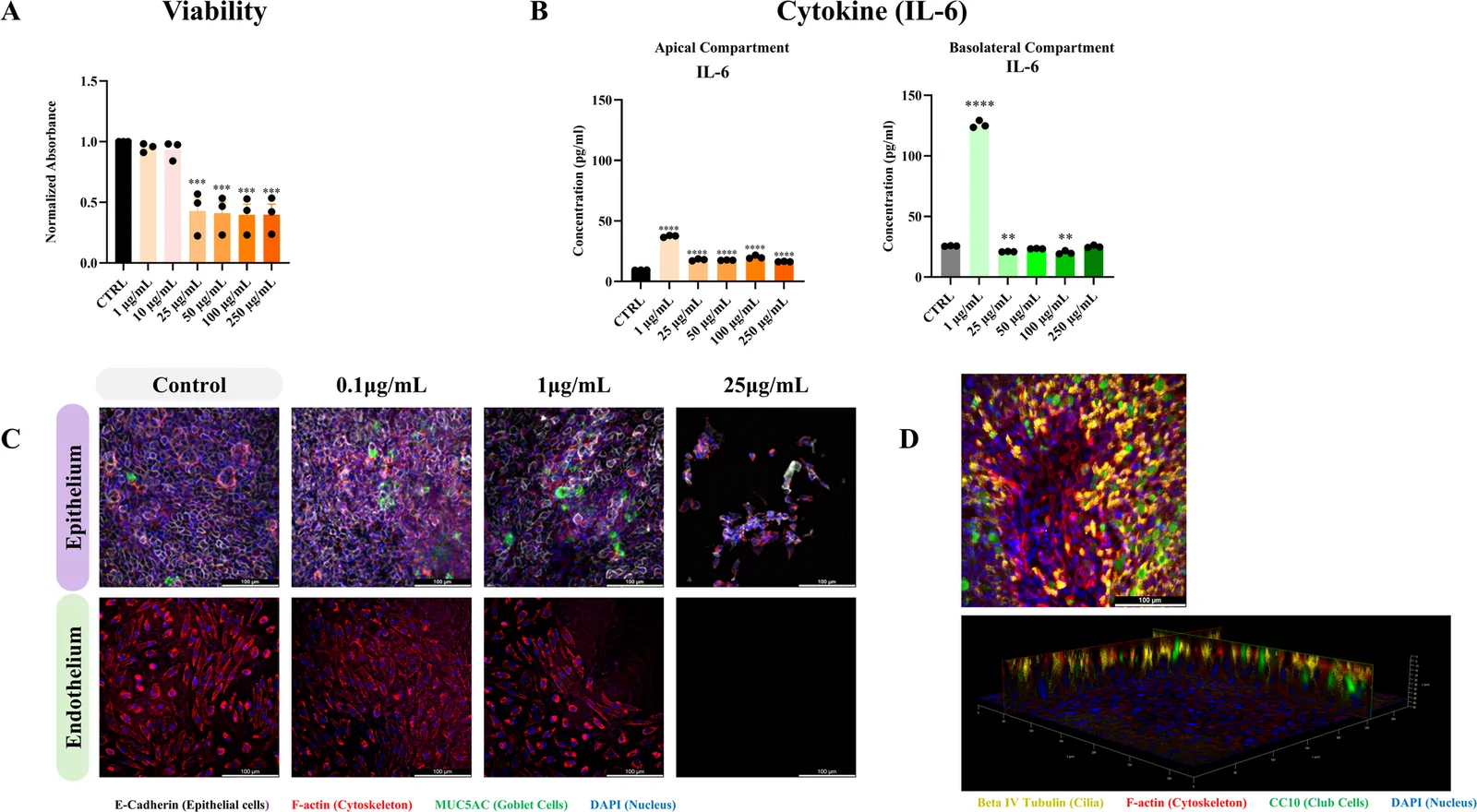
MIT induced cytotoxicity, inflammation, and barrier disruption in the NHBE/HUVEC co-culture system. (A) Viability of NHBE/HUVEC co-culture following MIT treatment (1–250 µg/mL) at various doses (data are presented as mean ± SEM, N=3, n=1). (B) IL-6 secretion measured by ELISA in apical and basolateral compartment following MIT treatment (1–250 µg/mL) (data are presented as mean ± SEM, N=3, n=1). (C) Immunofluorescence staining for epithelial cells (E-cadherin, white), cytoskeleton (F-actin), and goblet cells (MUC5AC) following MIT exposure. Scale bar = 100 µm. (D) Immunofluorescence staining for ciliated cells (beta-IV Tubulin) and club cells (CC10) after four weeks of differentiation at the ALI. Scale bar = 100 µm.

To investigate the effects of MIT on cellular-level toxicity, a tight and functional lung epithelial barrier was modeled using an NHBE/HUVEC co-culture system. In the co-culture model, cell viability was measured following 24 h of MIT treatment across a range of 0 µg/mL to 250 µg/mL (Fig. 1A). The cell viability remained unaffected at lower MIT concentrations (up to 10 µg/mL). A statistically significant reduction in viability was observed starting at the 25 µg/mL dose and higher concentrations. We also performed ELISA to evaluate the IL-6 response in the apical and basolateral compartments of our co-culture model following MIT treatment (Fig. 1B). In the apical compartment, IL-6 secretion was observed starting from 1 µg/mL of MIT, with higher doses showing a plateau in secretion levels. In contrast, the basolateral compartment exhibited significantly higher IL-6 secretion at 1 µg/mL. These results suggest a distinct pattern of IL-6 detection in the apical versus basolateral compartments, indicating a complex, dose-dependent inflammatory response to MIT exposure within the overall co-culture system.

MIT was administered at concentrations of 0.1 µg/mL, 1 µg/mL, and 25 µg/mL, and an immunofluorescence imaging analysis was performed to observe the expression of epithelial cells (E-cadherin), the cytoskeleton (F-actin), and mucus (MUC5AC) (Fig. 1C). As the concentration of MIT treatment increased, a greater loss of epithelial cells was observed in a dose-dependent manner. Similarly, endothelial cells exhibited cell detachment with increasing concentrations, showing a trend comparable to that of epithelial cell loss. At the highest concentration, both epithelial cells and nuclei displayed compromised morphology. Additionally, we observed that MUC5AC expression increased progressively with an increasing dose of MIT.

### Nebulized MIT induced cytotoxicity in an alveolar lung-on-chip co-culture model

This study evaluated the toxicity of MIT and PHMG using both 2D and 3D models of the alveolar barrier. In the 2D model, alveolar epithelial cells were cultured in standard 96-well plates to assess the cytotoxic response, and the test substances were nebulized for exposure. This approach revealed dose-dependent cytotoxic effects, with significant cell death occurring at higher MIT concentrations (Supplementary Figures S1A and S1B). MIT exhibited cytotoxicity even at 1 µg/mL, with a notable decrease in cell viability observed at 25 µg/mL. Brightfield imaging (Supplementary Figure S1C) further demonstrated distinct patterns of cellular disruption, with PHMG exposure resulting in widespread and more homogeneous barrier impairment, whereas MIT produced more localized effects. However, the 2D model presented limitations in its capacity to mimic the physiological lung environment. This could lead to elevated stress responses during exposure and may have influenced the observed cytotoxic outcomes.

To achieve a more physiologically relevant assessment, we employed an alveolar lung-on-chip incorporating breathing-like stretch motion, a biocompatible porous elastic substrate, co-culture with alveolar and endothelial cells, and ALI conditions. This system provided a more realistic simulation of the alveolar barrier and was used to evaluate the impact of aerosolized MIT on barrier integrity, with PHMG serving as a positive control due to its established cytotoxic effects on lung barrier integrity [13, 17].

**Fig. 2:**
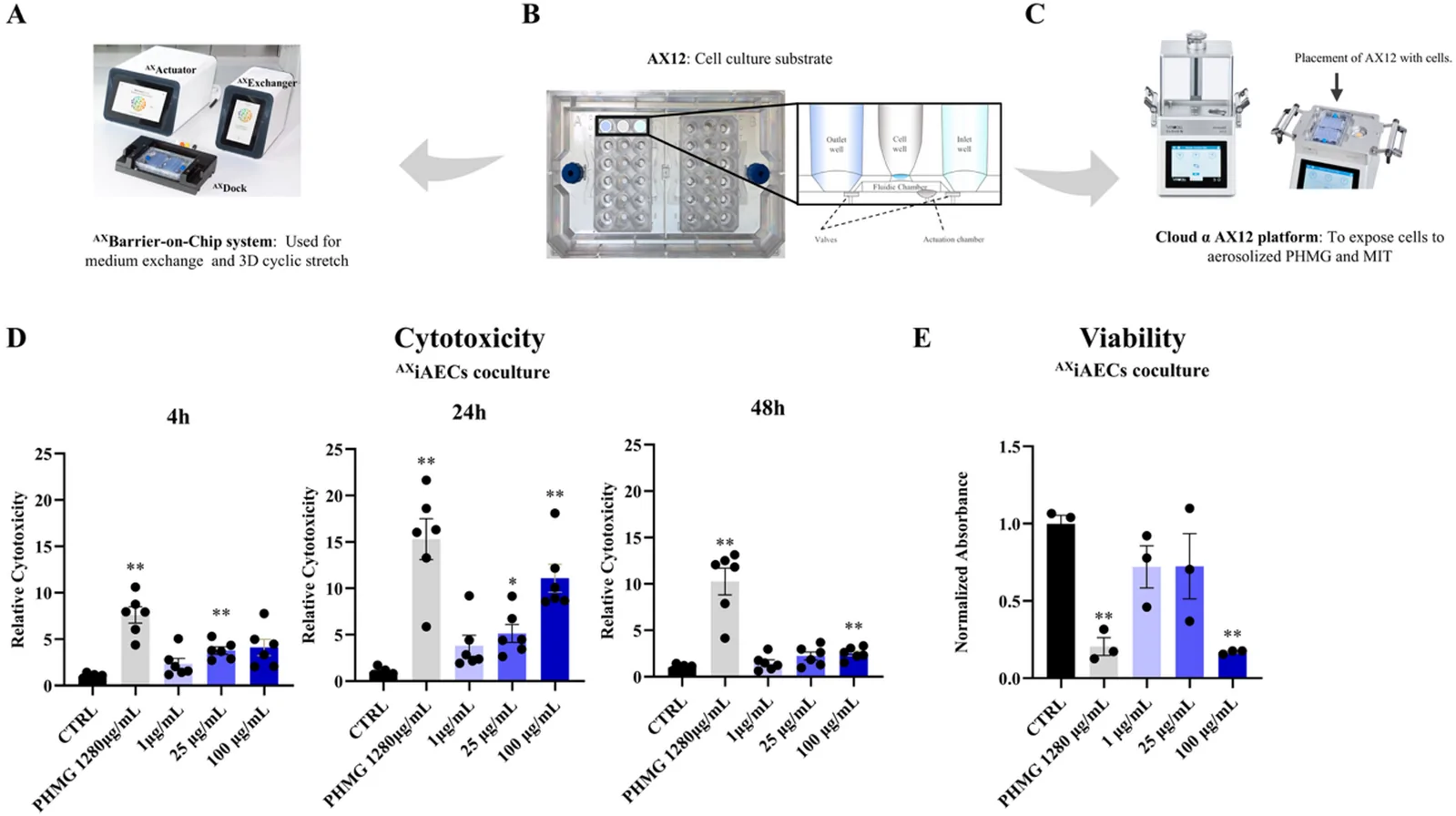
Experimental setup and effects of aerosolized MIT on cytotoxicity and viability in an alveolar co-culture model. **A** The different components of the ^AX^Barrier-on-Chip system to perform medium exchange and TEER measurement (^AX^Exchanger) and apply 3D cyclic stretch (^AX^Acctuator) on the AX12 (cell culture support). Image provided by AlveoliX. **B** A schematic illustration of the image provided by AlveoliX. **C** Left: In the Cloud α AX12 platform. Right: Placement of the cells seeded on the AX12 subjected to aerosolized exposure of the vehicle control, PHMG, and MIT. Image provided by VITROCELL Systems. **D** LDH release measurements at 4 h, 24 h, and 48 h following aerosolized exposure to vehicle control, PHMG (1280 µg/mL), and MIT (1–100 µg/mL) (data are presented as mean ± SEM, *N* = 1, *n* = 6). **E** Viability of alveolar cells following aerosolized MIT (1–100 µg/mL) and PHMG (1280 µg/mL) exposure (endpoint: cell viability; data are presented as mean ± SEM, *N* = 1, *n* = 3)

In the alveolar lung-on-chip, increased cytotoxicity was evident from the LDH measurements (Fig. 2D). At an MIT concentration of 25 µg/mL, nebulization resulted in approximately three-fold and five-fold increases in cytotoxicity at 4 h and 24 h, respectively. At an MIT concentration of 100 µg/mL, nebulization led to a dramatic increase in cytotoxicity at 24 h and 48 h. PHMG (1280 µg/mL) showed an even greater LDH release and the viability profile mirrored this trend. Notably, despite the much higher PHMG dose, the reduction in viability was comparable to that produced by MIT at 100 µg/mL (Fig. 2E).

**Fig. 3:**
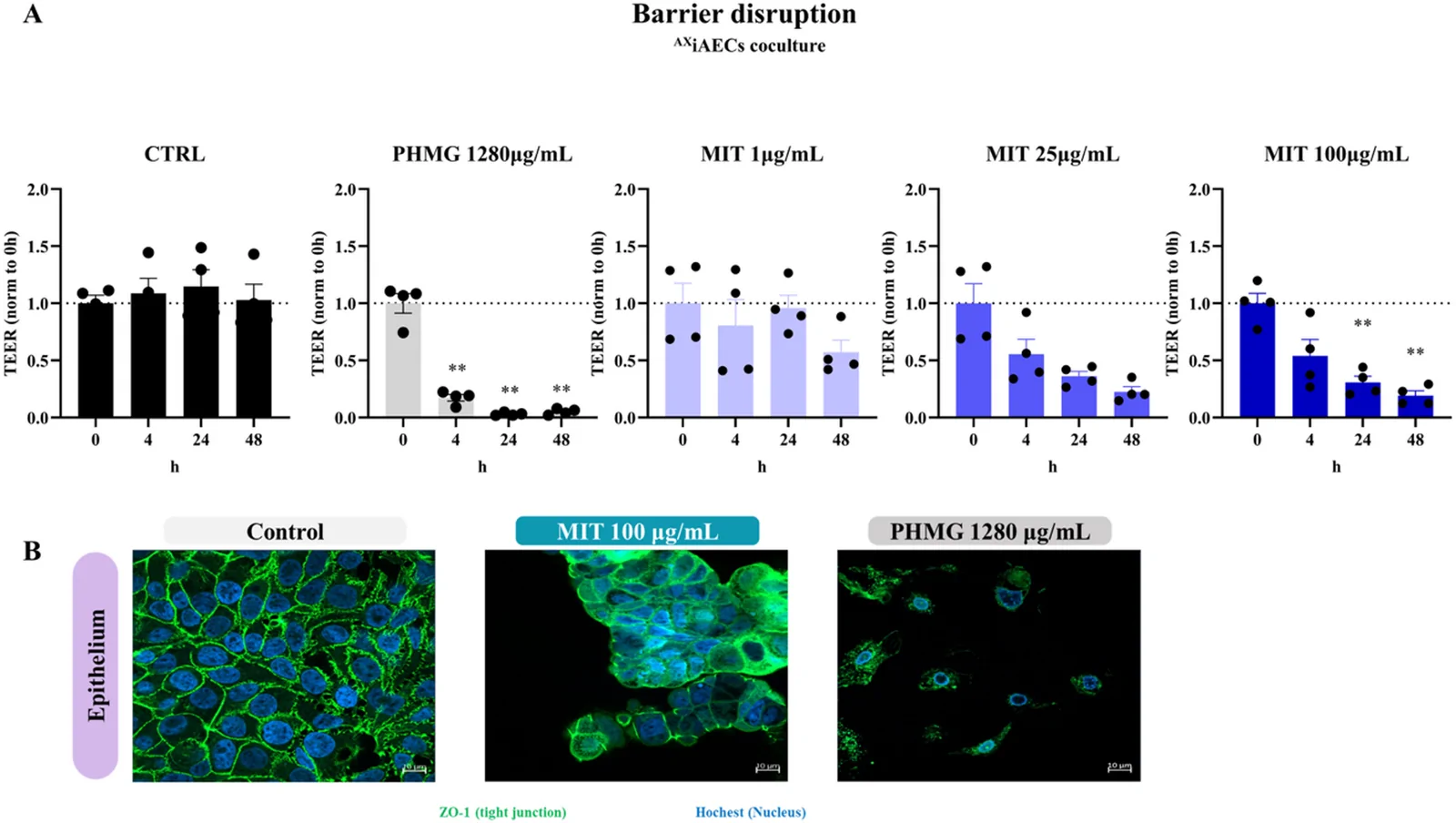
Effects of aerosolized MIT on barrier integrity in an alveolar lung-on-chip. **A** TEER measurements at 0 h, 4 h, 24 h, and 48 h are normalized to 0 h, showing the relative barrier disruption in response to PHMG and MIT compared to vehicle control (data are presented as mean ± SEM, *N* = 1, *n* = 4). **B** Immunofluorescence staining of alveolar cells post-aerosol exposure, highlighting tight junctions (ZO-1) and morphological changes. Scale bar = 10 μm

Consistent with this observation, the TEER values remained stable from 4 h to 48 h post-nebulization with the vehicle control under ALI + cyclic stretch conditions (Supplementary Figure S4), and when normalized to the baseline (0 h) (Fig. 3A).

MIT at 25 µg/mL exhibited a downward trend over time that did not reach statistical significance. Similarly, TEER measurements before (0 h) and after exposure revealed a statistically significant decrease in barrier integrity at MIT 100 µg/mL, with a drop to approximately 30% of the TEER compared to 0 h at 24 h and a drop to approximately 20% of TEER compared to 0 h at 48 h. PHMG (1280 µg/mL) reduced the TEER to near-baseline levels within 4 h, and this reduction persisted throughout the 48 h observation period.

At an MIT concentration of 100 µg/mL, nebulization led to a marked increase in cytotoxicity at 24 and 48 h. Consistent with the decrease in TEER, immunofluorescence staining revealed severe tight-junction disruption, island clusters of cells, and cell detachment (Fig. 3B).

The complementary use of 2D and 3D models enabled a comprehensive assessment of MIT and PHMG toxicity, with the 2D model serving as an efficient screening tool and the 3D model providing critical insights into mechanisms underlying toxicity in a more physiologically relevant setting, particularly with respect to barrier integrity and junctional disruptions under ALI conditions.

### Nebulized MIT induced cytotoxicity alveolar co-culture on chip

**Fig. 4:**
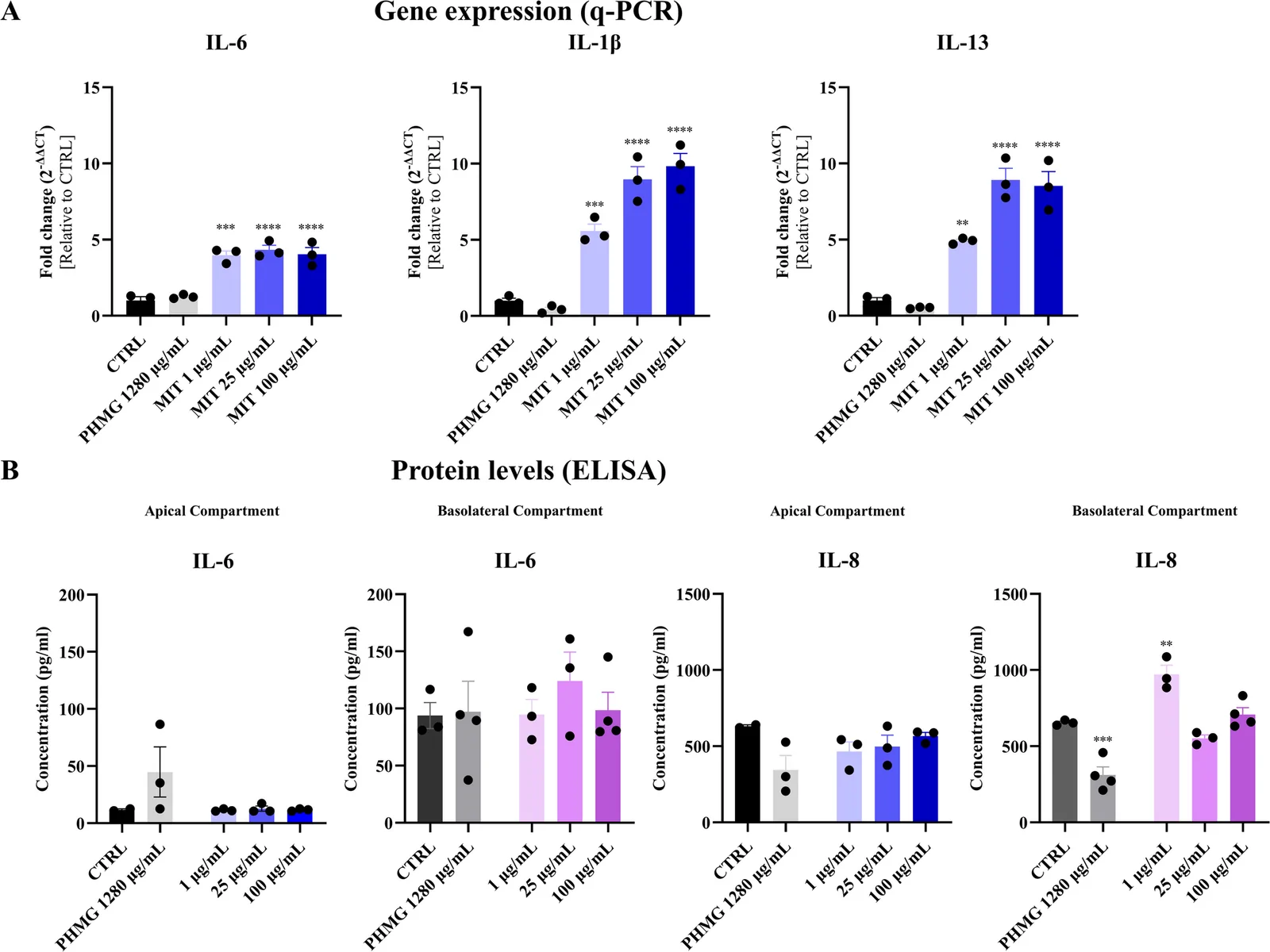
MIT- and PHMG-induced cytokine responses in alveolar epithelial cells. **A** mRNA expression levels of IL-6, IL-1β, and IL-13 analyzed by RT-qPCR following exposure to vehicle control, MIT, and PHMG under ALI and cyclic stretch. Gene expression levels are normalized to the vehicle control (data are presented as mean ± SEM, *N* = 1, *n* = 3). **B** IL-8 protein secretion measured by ELISA in alveolar epithelial cells following exposure to vehicle control, MIT, and PHMG. Results are presented as mean concentration (pg/mL) with statistical comparisons to the vehicle control (data are presented as mean ± SEM, *N* = 1, *n* = 2–4)

To assess the inflammatory response induced by MIT and PHMG, we analyzed the levels of the key cytokines, IL-6, IL-1β, IL-13, and IL-8, which are known to be critical mediators of inflammation. The results revealed that IL-8 secretion significantly increased in the basolateral compartment upon exposure to MIT at 1 µg/mL, compared to the vehicle control. In contrast, IL-6 protein levels showed no notable changes across any of the conditions. This suggests that IL-8 may play a more immediate role in the acute inflammatory response within this co-culture system (Fig. 4B).

Furthermore, the RT-qPCR analysis revealed that the mRNA levels of IL-6, IL-1β, and IL-13 were significantly increased across all concentrations of MIT (1 µg/mL, 25 µg/mL, and 100 µg/mL), demonstrating a consistent transcriptional inflammatory response independent of dose (Fig. 4A). Specifically, IL-6 mRNA levels increased 4.0-fold, 4.33-fold, and 4.01-fold at low, medium, and high MIT concentrations, respectively. IL-1β mRNA showed a greater increase, with 5.58-fold, 9.0-fold, and 9.8-fold upregulation across these concentrations. Similarly, IL-13 mRNA levels increased 4.1-fold, 7.5-fold, and 7.1-fold, confirming substantial activation of inflammatory gene expression in response to MIT.

Overall, these results highlight a nuanced inflammatory response to MIT and PHMG exposure: ELISA indicated an acute IL-8 response, whereas RT-qPCR revealed consistent cytokine gene activation across all MIT concentrations. Together, these analyses provide a comprehensive view of the inflammatory effects of MIT and PHMG on the alveolar barrier model.

## Discussion

This study highlights the urgent need to address MIT toxicity, particularly with regard to its use as a component of HDs. PHMG’s health impacts are currently well documented—severe pediatric interstitial lung disease linked to HD use prompted a ban on PHMG-containing products in Korea and Kim et al. (2014) reported a strong association between HD use, predominantly PHMG-p, and fatal interstitial lung disease in children. In contrast, MIT remains underexplored despite its role in severe lung conditions [18]. Our findings demonstrate the ability of MIT to disrupt epithelial barriers, reduce cell viability, and trigger inflammation in lung epithelial cells. Further research is thus necessary to clarify the toxic mechanism of MIT and inform public-health policies. For MIT, we used a stepwise range, starting from a physiologically plausible worst-case consumer exposure. Commercial CMIT/MIT products were sold at ~ 150 µg/mL before dilution, and realistic misuse (≈ 20:1 instead of the recommended 200:1) yields in-use concentrations of ~ 7–8 µg/mL [19, 20]. In a controlled humidifier-aerosol chamber study, a 6.5 ppm “normal-use” solution was aerosolized, and 24-hour adult lung deposition was modeled. This yielded a predicted daily alveolar surface dose of approximately 0.001 µg/cm², which closely matches the lowest deposition measured in our aerosol system (0.002 µg/cm²) [19]. Higher MIT doses were then included to define cytotoxic and inflammatory thresholds, consistent with reports that MIT alone causes dose-dependent epithelial injury at low µg/mL levels [11].

By contrast, PHMG was not employed to establish a minimal toxic dose but to serve as an acute-injury positive control. The concentration we used falls within the reported PHMG content of marketed humidifier disinfectant products (160–37,200 ppm) and reflects documented cases in which these products were used with much less dilution than instructed [19, 21]. The deposited surface dose (2.7 µg/cm²) was selected from published literature [13] as a toxicologically equivalent burden that compresses repeated high-intensity household exposure into an acute in vitro challenge. This is consistent with multi-week PHMG inhalation studies in rats showing that repeated exposure for 4 h/day, 5 days/week, over 3–4 weeks is sufficient to cause persistent epithelial barrier damage, alveolar inflammation, mucus hyperplasia, and fibrosis that continues even during recovery [22, 23].

To date, only animal studies have been conducted to evaluate the toxicity and biodistribution of CMIT/MIT. These studies revealed that 22% and 18% of the CMIT/MIT doses remained in the lung tissue seven days after intranasal and intratracheal exposure, respectively, demonstrating prolonged retention of the chemicals in the lungs. This retention correlated with increases in inflammatory cells in the BALF and histopathological changes, including fibrosis, inflammation, and mucus cell hyperplasia, along with collagen accumulation in the bronchial and alveolar regions [10]. As mentioned above, in an in vivo test using C57BL/6 mice, intratracheal or intranasal instillations of CMIT/MIT (≤ 1.14 mg/kg) for two weeks induced significant increases in alveolar inflammation, lung fibrosis, and mucous-cell hyperplasia [10]. However, such instillation bypasses physiological aerosol deposition, and interspecies differences in airway architecture and lung anatomy complicate the correlation of animal responses with human pathophysiological mechanisms. In retrospective epidemiology, the KCDC reviewed 530 suspected HDLI cases; among 36 exclusive CMIT/MIT users, three were HDLI-positive and one died, confirming a nontrivial risk despite a limited market share [1]. Nonetheless, the reliance on retrospective exposure estimation and mixed-product use limits accurate deposition-based dose-response assessment.

Notwithstanding these findings, animal studies do not fully replicate the human physiological environment [17]. To address this, we conducted a stepwise experimental approach that started with primary lung cells and progressed to a lung-on-chip for a more human-relevant assessment [24]. The concentrations used in this study (1–250 µg/mL) are significantly lower than the highest dose (1,140 µg/mL) reported in animal studies and align with the lower range of toxicological exposure (40 µg/mL). These concentrations are designed to assess direct cellular responses and allow for translational comparisons with in vivo studies [10].

Until recently, upper-airway studies of MIT have relied mainly on rodent inhalation models and on submerged cultures of immortalized bronchial epithelial cells such as BEAS-2B cells grown in Transwell inserts, leaving fully differentiated primary NHBE cells cultured under ALI conditions unexplored [10–12]. In our study, Transwell-based NHBE/HUVEC co-cultures were used to assess upper-airway toxicity. MUC5AC, which is often upregulated in response to epithelial injury and inflammation, was analyzed as a marker of mucus cell hyperplasia. Dose-dependent reductions in cell viability, increased MUC5AC expression, and elevated IL-6 secretion were observed, and the results were used to design subsequent experiments. Notably, MUC5AC overexpression and IL-6 secretion were observed following MIT treatment in this study, showing a trend similar to the previously reported PHMG-induced MUC5AC upregulation and IL-6 secretion [25, 26]. In this study, IL-6 secretion was elevated in both the apical and basolateral comparts compared with that in the control group, but it did not follow a strictly dose-dependent pattern, with the highest secretion observed at 1 µg/mL. This nonlinear behavior aligns with previous findings that cytokine responses often plateau, rather than continuing to rise, at higher concentrations [27]. Immunofluorescence images revealed significant cell detachment and membrane disruption starting at 25 µg/mL, suggesting that cellular damage may influence IL-6 secretion. Although these experiments began with NHBE cultures differentiated for four weeks at ALI, MIT challenge was performed under 24 h submerged apical dosing rather than aerosol delivery directly onto the ALI surface. As a result, the bronchial data should be interpreted as an acute direct-contact injury model rather than a fully physiological aerosol-at-ALI exposure.

To overcome the limitations of in vivo studies, we designed a physiologically relevant inhalation exposure system to more accurately assess MIT toxicity. After obtaining the bronchial model findings, we advanced to a distal-lung alveolar model via the Cloud α AX12 platform to evaluate lower airway toxicity more physiologically. To bridge this gap, we employed the ^AX^Barrier-on-Chip system incorporating cyclic stretch aerosolization of MIT at concentrations of 1, 25, and 100 µg/mL, resulting in surface deposition doses of 0.002, 0.053, and 0.212 µg/cm², respectively. In 2D cultures, cell viability decreased below 50% at 25 µg/mL, whereas the 3D alveolar lung-on-chip maintained viability above 50% up to 100 µg/mL. This suggests increased tolerance due to physiological stretching and multicellular interactions. Nevertheless, within 48 h, the alveolar lung-on-chip exhibited significant epithelial barrier disruption and elevated inflammatory cytokine levels, resembling acute responses preceding chronic fibrosis in rodent models. By integrating human-derived cells, authentic aerosol exposure, dynamic breathing mechanics, and precise surface-dose quantification, this platform mitigates species-specific discrepancies, delivery-mode artifacts, and exposure misclassification. These findings support the utility of stretchable lung-on-chip systems as prevalidation bridges between animal studies and human clinical outcomes.

To better simulate in vivo-like physiological exposure conditions, we progressed to an alveolar lung-on-chip model using the Cloud α AX12 platform. This advanced inhalation in vitro platform has been validated for assessing aerosolized PHMG toxicity and demonstrates that 3D exposure systems combined with ALI and stretch conditions more effectively mimic the physiological lung microenvironment compared to traditional 2D models [13].

On the basis of these findings, we utilized PHMG as a positive control in our study. In the ^AX^Barrier-on-Chip system, the alveolar barrier was simulated more physiologically under ALI conditions, with endothelial co-culture and breathing motion. TEER analysis and immunofluorescence results demonstrated that both MIT (100 µg/mL) and PHMG (1280 µg/mL) compromised barrier integrity, with MIT exhibiting particularly significant effects after 24 h. At 1280 µg/mL, PHMG caused a rapid and substantial decrease in TEER, along with tight junction disruption and cell detachment, as observed through brightfield imaging and immunofluorescence. These findings, which are consistent with results from other studies, indicate that PHMG acts through mechanisms that rapidly and severely compromise the structure and function of the lung epithelial barrier [23]. For MIT, the cytotoxicity significantly increased at MIT concentrations of 25 µg/mL and 100 µg/mL between 4 and 24 h, reaching a peak at the 24-hour time point. At 48 h, the cytotoxicity signal was observed to decrease compared to the 24-hour levels.

To evaluate the inflammatory response, ELISA and RT-qPCR analyses were performed. The results revealed that MIT (1 µg/mL) significantly increased IL-8 secretion in the basolateral compartment, similar to our positive control PHMG (1280 µg/mL), suggesting the critical role of IL-8 in acute inflammatory response. In contrast, the IL-6 protein levels did not significantly change. This suggests that within the basolateral compartment of the alveolar lung-on-chip, the initial inflammatory response to both substances may primarily involve IL-8 more than IL-6 at the protein level. While human endothelial cells are known to release IL-8 during inflammation [24], the source of the IL-8 detected in the basolateral compartment cannot be solely attributed to endothelial cells due to potential diffusion. Nonetheless, the pronounced increase in IL-8 in this compartment indicates a significant inflammatory response occurring at or transmitted across the alveolar barrier.

Meanwhile, the RT-qPCR analysis demonstrated that MIT exposure (1, 25, 100 µg/mL) significantly upregulated the mRNA levels of IL-6, IL-1β, and IL-13 in a concentration-dependent manner. This suggests a distinct transcriptional inflammatory mechanism compared to PHMG, which resulted in the absence of significant changes in cytokine gene expression. These findings are consistent with previous studies demonstrating that CMIT/MIT exposure induces increases in IL-6 and IL-13 in mouse models of respiratory toxicity and allergic dermatitis, as well as an increase in IL-1β in alveolar epithelial cell models, and they underscore the involvement of these cytokines in inflammation and fibrosis pathways [28–30]. Notably, the significant upregulation of IL-6 mRNA did not correlate with secreted protein levels (Fig. 4B). This appears to be a selective biological response, as a robust protein signal for IL-8 was detected in the same samples.

This study demonstrates the toxic potential of MIT to induce epithelial-barrier disruption, inflammation, and fibrosis, emphasizing its role in respiratory damage. By combining two complementary in-vitro strategies—a bronchial Transwell co-culture model and an alveolar lung-on-chip co-culture model—we highlight the value of physiologically relevant models for assessing inhaled toxicants. The Transwell co-culture model employs well-differentiated primary NHBE cells dosed apically under submerged conditions, whereas the alveolar co-culture model—seeded onto a biocompatible porous membrane—undergoes cyclic stretching and receives aerosolized MIT. These differences in cell type, substrate, mechanical load, and administration route account for the obtained complementary toxicodynamic profiles. The pronounced upregulation of inflammatory mediators (IL-6, IL-1β, IL-13) and MUC5AC provides mechanistic insights into MIT-induced chronic inflammation and mucus hyperplasia. Supplementary Figure S3 provides a schematic illustration of the experimental setup.

Collectively, our findings establish a foundation for refining risk assessments and public-health policies aimed at mitigating the impact of HD components—particularly MIT—on human respiratory health. Although recent court rulings in South Korea acquitted manufacturers of CMIT/MIT-containing disinfectants owing to an alleged lack of scientific evidence linking these biocides to lung injury [31], our biodistribution and toxicity data suggest that this conclusion warrants careful reconsideration.

Future studies should focus on elucidating how MIT affects alveolar regions, including in particular its role in disrupting epithelial barrier integrity and inducing inflammatory cytokine responses. The observed overexpression of MUC5AC in NHBE cells highlights the influence of MIT on mucus production in the bronchial region, while alveolar-specific effects underscore the capacity of MIT to provoke inflammation and barrier disruption. To evaluate region-specific toxicity more comprehensively, advanced in vitro models integrating NHBE-derived bronchial epithelium, ALI cultures with surfactant components (SP-B and SP-C), and immune cells such as macrophages are needed.

Long-term exposure studies under physiologically relevant conditions, combined with multiomics approaches, will help clarify the broader pathophysiological effects of MIT. To fully characterize the toxicity profiles of MIT and CMIT/MIT, future research should also explore chronic exposure, recovery dynamics, and fibrotic remodeling. The incorporation of advanced readouts such as single-cell transcriptomics and high-content imaging will yield mechanistic insights into cell-type-specific injury and repair. As the inhalation of chemical biocides remains a pressing public health concern, human-relevant in vitro models are essential to bridge the gap between experimental findings and regulatory decision-making. Ultimately, integrating these platforms into standard risk assessment frameworks could improve predictive accuracy and support the safe use and regulation of inhalable consumer products.

## Conclusion

In conclusion, this study integrated 2D (96-well plate) and 3D (Transwell and alveolar lung-on-chip) in vitro platforms to assess region-specific lung toxicity of MIT compared with PHMG. While PHMG served as a known positive control, MIT caused pronounced epithelial barrier disruption, inflammatory cytokine induction, and MUC5AC overproduction—notably occurring at much lower concentrations than PHMG. These findings underscore the potential of MIT to contribute to HD-associated lung injury. By employing ALI culture, aerosol exposure, and dynamic stretching, the alveolar lung-on-chip model provides a physiologically relevant assessment that overcomes key limitations of animal models and static in vitro assays. Our findings reveal distinct, dose- and time-dependent toxicity profiles for MIT and support the use of advanced human-relevant models in regulatory toxicology. Continued research involving long-term exposure and immune cell co-culture will be critical to fully characterize chronic responses and guide public health policy on the safe use of inhalable biocides.

## Supplementary Information

Supplementary Material 1.

## Data Availability

All data generated or analyzed during this study are included in this published article [and its supplementary information files].

## Abbreviations

ABM: Alveolar Barrier Medium
ALI: Air–Liquid Interface
^AX^iAECs: AlveoliX immortalized Alveolar Epithelial Cells
hLMVECs: AlveoliX human Lung Microvascular Endothelial Cells
BALF: Bronchoalveolar Lavage Fluid
BSA: Bovine Serum Albumin
CCK8: Cell Counting Kit-8
CMIT: 5-Chloro-2-methyl-4-isothiazolin-3-one
CS: Cyclic Stretch
DPBS: Dulbecco’s Phosphate-Buffered Saline
EGM: Endothelial Cell Growth Medium
ELISA: Enzyme-Linked Immunosorbent Assay
EVOM: Epithelial Volt/Ohm Meter
F-actin: Filamentous Actin
HD: Humidifier Disinfectant
HDLI: Humidifier Disinfectant-Associated Lung Injury
HUVEC: Human Umbilical Vein Endothelial Cell
IL: Interleukin
LDH: Lactate Dehydrogenase
MIT: 2-Methyl-4-isothiazolin-3-one
MUC5AC: Mucin 5AC
NHBE: Normal Human Bronchial Epithelial cell
PGH: Oligo(2-(2-ethoxy)ethoxyethyl) Guanidinium Chloride
PHMG: Polyhexamethylene Guanidine
qPCR: Quantitative Polymerase Chain Reaction
RT: Room Temperature
RT-qPCR: Reverse Transcription Quantitative Polymerase Chain Reaction
SEM: Standard Error of the Mean
TEER: Trans-Epithelial Electrical Resistance
ZO-1: Zonula Occludens-1
