# Supplementary material for: Evaluation of 2-methyl-4-isothiazolin-3-one-induced human pulmonary toxicity using integrated air-liquid-interface and a lung-on-chip
Source: Respir Res. 2026 May 27;27:329. doi: 10.1186/s12931-026-03733-z (PMC13508333; doi:10.1186/s12931-026-03733-z)
Supplement: Supplementary file 1 [file 12931_2026_3733_MOESM1_ESM.docx]

**FIGURE. S1. MIT-induced barrier disruption and cytotoxicity in an alveolar culture model in 2D**

**FIGURE. S1. MIT-induced barrier disruption and cytotoxicity in an alveolar culture model in 2D.** (A) Cytotoxicity measurements at 24 h post-aerosolization with vehicle control, PHMG, and MIT (data are presented as mean ± SEM, N=1, n=4-6). (B) Cell viability measurements at 24 h post-aerosolization with vehicle control, PHMG, and MIT (data are presented as mean ± SEM, N=1, n=4-12). (C) Brightfield imaging of alveolar cells at 24 h post-aerosolization with vehicle control, PHMG, and MIT. Cellular disruption, detachment, and barrier degradation were observed following PHMG and MIT exposure. Scale bar = 275 µm.

**FIGURE. S2. p values for figures**

| **Figure Number** | **p values** |
| --- | --- |
| 1-A | 25 µg/mL: p = 0.0004; 50 µg/mL: p = 0.0003; 100 µg/mL: p = 0.0002; 250 µg/mL: p = 0.0002 vs. CTRL |
| 1-B | Apical Compartments: 1 µg/mL, 25 µg/mL, 50 µg/mL, 100 µg/mL, and 250 µg/mL: all *p* < 0.0001 vs. CTRL  Basolateral Compartments: 1 µg/mL: p < 0.0001; 25 µg/mL: p = 0.0071; 100 µg/mL: p = 0.0024 vs. CTRL |
| 2-B | 4hr: PHMG 1280 µg/mL: p = 0.0024; 25 µg/mL: p = 0.0018 vs. CTRL  24hr: PHMG 1280 µg/mL: p = 0.0045; 25 µg/mL: p = 0.0287; 100 µg/mL: p = 0.0039 vs. CTRL  48hr: PHMG 1280 µg/mL: p = 0.0046; 100 µg/mL: p = 0.0049 vs. CTRL |
| 2-C | PHMG 1280 µg/mL: p = 0.0026; 100 µg/mL: p = 0.0019 vs. CTRL |
| 3-A | PHMG: 4 h: p = 0.0021; 24 h: p = 0.0036; 48 h: p = 0.0039  MIT 100 µg/mL: 24 h: p = 0.0030; 48 h: p = 0.0030 vs. 0 h |
| 4-A | IL-6:1 µg/mL: p = 0.0001; 25 µg/mL: p < 0.0001;100 µg/mL: p < 0.0001 vs. CTRL  IL-1β: 1 µg/mL: p = 0.0008; 25 µg/mL: p < 0.0001; 100 µg/mL: p < 0.0001 vs. CTRL  IL-13: 1 µg/mL: p = 0.0018; 25 µg/mL: p < 0.0001; 100 µg/mL: p < 0.0001 vs. CTRL |
| 4-B | PHMG 1280 µg/mL: p = 0.0005; 1 µg/mL: p = 0.0017 vs. CTRL |
| S-1 A | 1 µg/mL: p = 0.0017; 10 µg/mL: p = 0.0379; 50 µg/mL: p = 0.0005; 100 µg/mL: p = 0.0127 vs. CTRL |
| S-1 B | PHMG 1280 µg/mL: p = 0.0002; 25 µg/mL: p = 0.0061; 50 µg/mL: p = 0.0004; 100 µg/mL: p = 0.0015; 250 µg/mL: p = 0.0002 vs. CTRL |

**FIGURE. S3. For schematic illustration of the experimental setup**

**FIGURE S4. Effects of aerosolized MIT on TEER**

**FIGURE S4. Effects of aerosolized MIT on TEER.** Raw TEER (Ω⋅cm^2^) measurements at 0 h, 24 h, and 48 h showing barrier disruption in response to vehicle control, PHMG, and MIT (data are presented as mean ± SEM, N=1, n=4).
